# Integration of assisted partner services within Kenya’s national HIV testing services program: A qualitative study

**DOI:** 10.1371/journal.pgph.0001586

**Published:** 2023-02-10

**Authors:** Beatrice M. Wamuti, Mercy Owour, Chris Obong’o, Wenjia Liu, Edward Kariithi, Harison Lagat, George Otieno, Monisha Sharma, David A. Katz, Sarah Masyuko, Carey Farquhar, Bryan J. Weiner

**Affiliations:** 1 Department of Global Health, University of Washington, Seattle, Washington, United States of America; 2 Independent Consultant, Nairobi, Kenya; 3 PATH- Kenya, Kisumu, Kenya; 4 School of Nursing, University of Washington, Seattle, Washington, United States of America; 5 National AIDS and STI Control Program, Ministry of Health, Nairobi, Kenya; 6 Department of Epidemiology, University of Washington, Seattle, Washington, United States of America; 7 Department of Medicine, University of Washington, Seattle, Washington, United States of America; University of the Witwatersrand, SOUTH AFRICA

## Abstract

Assisted partner service (aPS) augments HIV case-finding among sex partners to individuals newly diagnosed with HIV. In 2016, aPS was incorporated into the national HIV testing services (HTS) program in Kenya. We evaluated the extent of, barriers to, and facilitators of aPS integration into HTS. We conducted semi-structured in-depth interviews (IDIs) with 32 stakeholders selected using purposive sampling at national, county, facility, and community levels. IDIs were conducted at two timepoints, at baseline from August-September 2018 in Kisumu and January-June 2019 in Homa Bay, and at follow-up from May-August 2020 to understand changes in aPS integration over time. We defined integration as the creation of linkages between the new intervention (aPS) and the existing HTS program. Data were analyzed using thematic content analysis. We found varying degrees of aPS integration, highest in procurement/logistics and lowest in HTS provider recruitment/training. At baseline, aPS integration was low and activities were at an introductory phase. At follow-up, aPS was integrated in almost the entire HTS program with the exception of low community awareness, which was noted at both baseline and follow-up. There was increasing routinization with establishment of clear aPS cycles, e.g., quarterly data review meetings, annual budget cycles and work-plans. Major barriers included limited government funding, staff constraints, and inadequate community-level sensitization, while key facilitators included increased resources for aPS, and community health volunteer (CHV) facilitated awareness of aPS. Varying degrees of aPS integration across different units of the national HTS program highlights challenges in funding, human resource, and public awareness. Policymakers will need to address these barriers to ensure optimal provision of aPS.

## Introduction

There are approximately 38.4 million living with HIV (PLWH), of whom 5.9 million (15%) are unaware of their HIV-positive status [1]. HIV case-finding is the first step in offering preventive messaging, referral and linkage to HIV care and treatment services for PLWH, and includes such interventions as assisted partner services (aPS) [2]. aPS has been used to augment HIV case-finding and involves tracing, testing and linking to care sex and drug-injecting partners of individuals diagnosed with HIV [2]. aPS was first recommended in 2016 by the World Health Organization (WHO) and included as part of the HIV testing services (HTS) package [2]. In many African countries, aPS is being promoted through ministries of health (MOHs) as it supports the UNAIDS 95-95-95 targets i.e., 95% of people living with HIV know their HIV status, 95% of people who know their status are receiving treatment, and 95% of people on HIV treatment achieve viral suppression [3–8].

After a successful cluster-randomized controlled trial in Kenya that found aPS to be effective, safe and cost-effective, the Kenyan MOH incorporated aPS into its national HTS program [9–12]. It is known that new interventions that were efficacious in controlled trials may have reduced effectiveness in real-life settings [13]. Also, most aPS literature is from controlled trials or pilot programs with a paucity of studies systematically evaluating national scale-up of the program [5,14–20]. These evaluations of pilot projects in Tanzania [21] and program-level integration in Cameroon [5] and Mozambique [14] do not describe how to effectively integrate the intervention into national HTS programs.

Integration, defined as the creation of linkages between new interventions and existing programs to improve healthcare delivery, occurs across institutional subsystems, defined as operational divisions within the healthcare system responsible for HTS e.g., procurement logistics, and service delivery [22–24]. For seamless integration of aPS to occur, it is important to identify key barriers and facilitators and manage them appropriately. Studies exploring the integration of new interventions into HTS programs have generally focused on service delivery at the facility level and not within subsystems at the national level, while none have evaluated the extent of integration over time [25]. In a study evaluating the integration of HIV pre-exposure prophylaxis (PrEP) in Kenya, authors focused on process flows at the facility level [26] while in a systematic review of family planning integration into HIV programs, most studies focused on client outcomes with few focusing on policy level implementation processes [27].

In our study, we explored the extent of, barriers to, and facilitators of aPS integration into the national HTS program in Kenya. This was conducted as part of a larger project evaluating the effectiveness of aPS implementation in western Kenya. We qualitatively described the extent of aPS integration across key MOH subsystems and compared the levels of aPS integration over time.

## Methods

### Study design

Qualitative in-depth interviews (IDIs) were nested within the aPS scale-up study, a collaboration between MOH, PATH-Kenya, and the University of Washington, described in detail elsewhere [28]. The study took place in 31 healthcare facilities in Kisumu and Homa Bay, two counties in western Kenya with the highest HIV prevalence in the country [29]. Briefly, women newly testing HIV-positive (female index clients) were enrolled to the study and details on their male sex partners was collected. HTS providers then traced and notified these partners of their potential HIV exposure and offered HIV testing. Partners testing HIV positive were referred for HIV care and treatment, while those testing HIV negative received counselling on HIV prevention.

### Study participants

Using purposive sampling, we conducted 40 interviews (twenty each at baseline and follow-up) with 32 key aPS stakeholders at the national, county, facility, and community levels. Eight of these individuals were interviewed twice by virtue of their job positions. This sample was selected to include perspectives from all healthcare system levels in Kenya and provide a holistic perspective of aPS integration. At the national level, we interviewed MOH policy makers at the National AIDS and STI Control program (NASCOP) and at implementing partner organizations. NASCOP is the main governmental institution in charge of HTS in Kenya while implementing partner organizations are typically large non-governmental organizations offering technical assistance and implementation support for HTS.

At the county level, we interviewed county and sub-county AIDS coordinators (CASCOs, sub-CASCOs) who are NASCOP staff who translate national HTS policy at the counties and sub-counties, respectively. At the facility level, we interviewed facility-in-charges who are government workers supervising individual healthcare facilities and supporting policy implementation and service delivery. At the community level, we interviewed community representatives who are trusted community members appointed by local leaders as liaisons between their communities and the healthcare facilities. They were selected after discussions with facility-in-charges and PATH-Kenya staff to gain community perspectives on aPS integration. We included this group during the follow-up interviews when it became apparent at baseline that we were missing the voices of the communities receiving aPS.

### Conceptual framework

**Fig 1:**
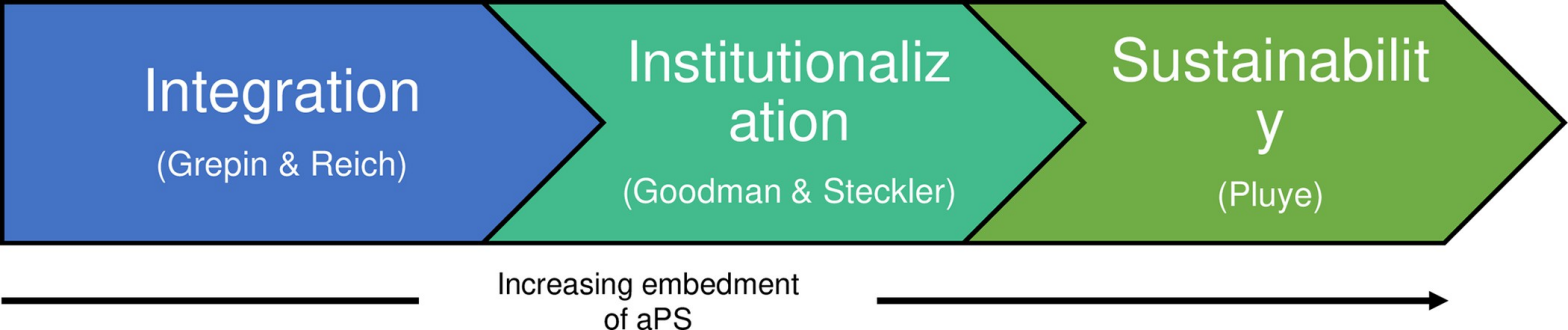
Conceptual framework of aPS integration in Kenya’s HTS program.

**Fig 2:**
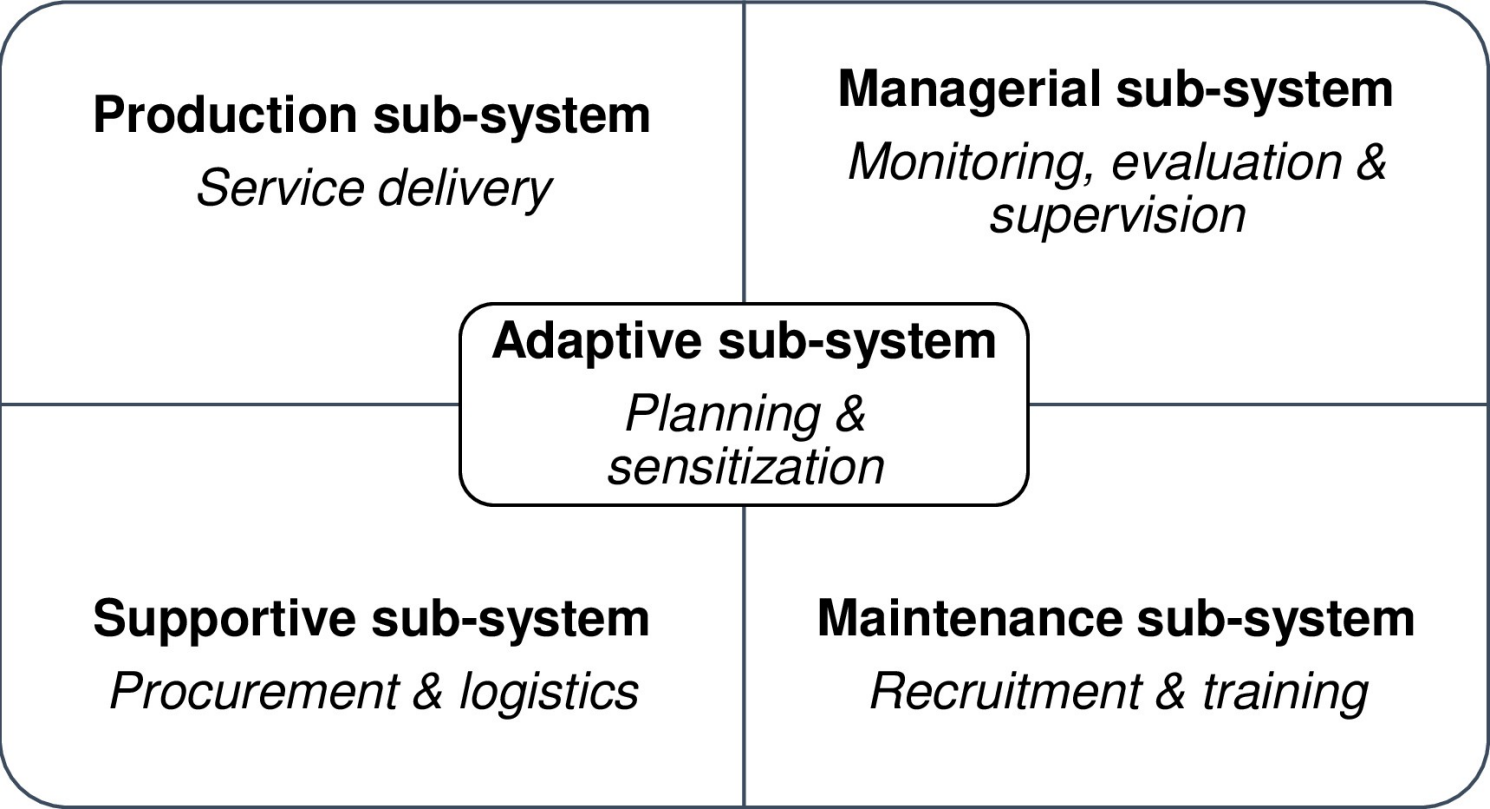
Organizational subsystems in Kenya’s HTS program based on conceptual framework.

We developed our conceptual framework after extensive review of literature on integration of new interventions into national programs, and combined concepts from three theoretical frameworks, namely integration by Grepin & Reich, institutionalization by Goodman & Steckler, and sustainability by Pluye (*Figs 1 and 2)* [22,24,30]. Integration was defined as the creation of linkages between aPS and the existing HTS program [22]; institutionalization was defined as the viability of aPS [24]; and sustainability was defined as the long-term continuation of aPS in HTS [30].

Integration was operationalized in three ways: full, partial, or minimal integration, referring to complete, incomplete, or limited aPS incorporation to HTS, respectively [22].

Institutionalization was operationalized using a two-dimensional matrix of organizational subsystems and levels of institutionalization [24]. We defined five organizational sub-systems: 1) adaptive subsystem that modifies MOH’s organizational structure and processes to allow for aPS; 2) supportive subsystem that procures resources for aPS provision; 3) maintenance subsystem that reinforces the roles of aPS staff; 4) production subsystem that delivers aPS through HTS providers by offering partner elicitation, tracing, and notification services; and 5) managerial subsystem that reviews key aPS indicators. We also defined three levels of institutionalization: passages that describe the introduction of aPS into HTS, routinization that describes its recurrent adoption through cycles e.g., budget cycles, and niche saturation that refers to long-term aPS provision within HTS.

Sustainability was operationalized in three ways: weak sustainability–absence of aPS routines, medium sustainability–presence of non-standard aPS routines e.g., activities not supported through government endorsed tools, and strong sustainability—presence of aPS that are supported at state level e.g. through government supported aPS guidelines [30].

### Study procedures and data collection

Key aPS stakeholders were approached to participate in IDIs, each lasting one to two hours. IDIs were conducted at baseline and follow-up to better understand perceived changes in aPS integration over time. Baseline interviews were conducted in two phases; first, from August-September 2018 in Kisumu, and second from January-June 2019 in Homa Bay as its facilities entered the study five months after the start of the Kisumu baseline interviews. Follow-up interviews occurred approximately one and a half years later from May-August 2020 in both counties.

Semi-structured interview guides based on the conceptual framework were administered (S2 and S3 Files). IDIs were conducted either in person or on phone in either English, Swahili, or Luo by two qualified qualitative interviewers (baseline: CO, follow-up: MO), neither of whom were known to the respondents. While the baseline interviews were conducted in person, all the follow-up interviews were conducted on phone due to COVID-19 social distancing restrictions. IDIs were audio-recorded and transcribed by a transcriber familiar with the three languages. The qualitative interviewers verified all transcripts and corrected any inconsistencies.

### Data analysis

Personal identifiers were removed from recorded interviews and corresponding transcripts which were then assigned identification numbers. CO, MO, and BMW used deductive and inductive coding to develop the codebook based on structured codes from the conceptual framework and emerging codes from the interviews. We identified 112 codes that were organized into 10 themes and 34 sub-themes. The codebook was pilot tested on two transcripts and revised. Researchers then coded the remaining transcripts independently, reviewed each other’s coded transcripts, and reached consensus through discussion. Interview transcripts were analyzed using thematic content analysis and coders wrote memos to organize conceptual categories and track emerging insights on the data. Demographic characteristics of the interviewees, selected excerpts of interview quotes, and the degree of integration have been presented using tables and quotes. Analyses were conducted using ATLAS.Ti version 8.4.4 and Microsoft Excel.

### Ethical considerations

This study received ethical approval from the Kenyatta National Hospital–University of Nairobi Ethics and Research Committee (P465/052017) and the University of Washington Institutional Review Board (STUDY00002420). All interviewees provided written informed consent prior to participation.

## Results

Sociodemographic characteristics for the 32 aPS stakeholders are described in **Table 1**. Five MOH policy makers, 10 HIV implementing partner organization representatives, five CASCO/SCASCOs, eight facility-in-charges, and four community representatives were interviewed. Most interviewees had post-secondary education, and there was an almost equal number of male and female interviewees.

**Table 1:** Stakeholder characteristics.

Characteristic	Detail	Total
Role	MOH policy maker	5 (16%)
	HIV implementing partner	10 (31%)
	CASCO^1^	2 (6%)
	SCASCO^2^	3 (9%)
	Facility-in-charge	8 (25%)
	Community representative	4 (13%)
Gender	Female	14 (44%)
	Male	18 (56%)
Region	National	9 (28%)
	Homa Bay	13 (41%)
	Kisumu	10 (31%)
Education	Some primary education	1 (3%)
	Secondary education	1 (3%)
	Post-secondary education	30 (94%)

^1^CASCO–County AIDS and STI (STI–Sexually transmitted infection) coordinator;

^2^SCASCO—Sub-county AIDS and STI coordinator. Eight individuals were interviewed twice (MOH policymaker = 1, CASCO = 1, SCASCO = 3, facility-in charges = 3).

### aPS integration, institutionalization, and sustainability by HTS subsystem

**Fig 3:**
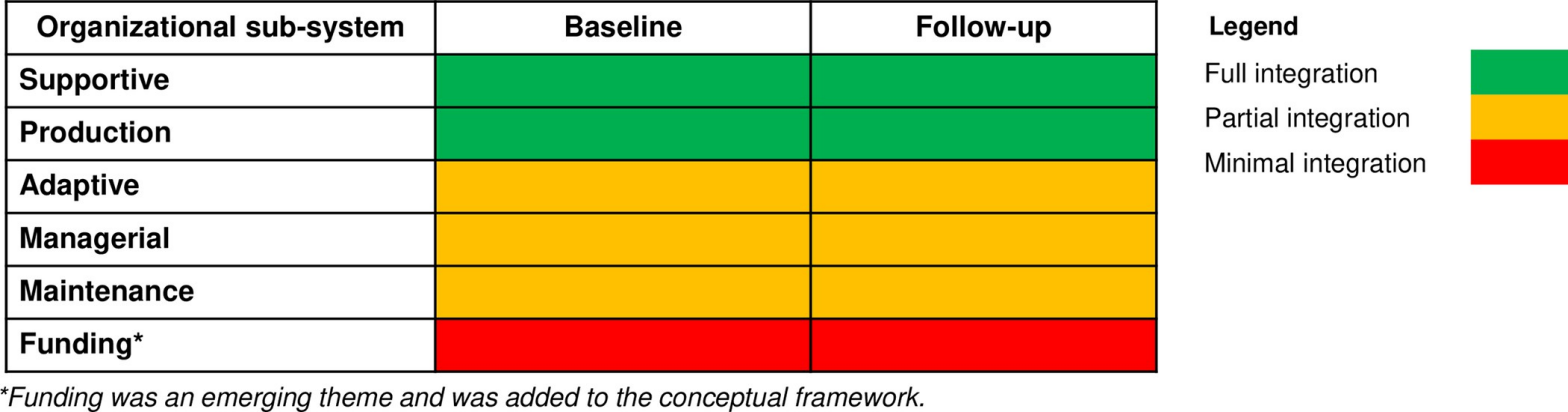
Extent of aPS integration across organizational subsystems.

At baseline, aPS had been fully integrated into the national HTS procurement and logistics systems (supportive subsystem) and this persisted into program roll-out. This included the routine supply of medical supplies and commodities to support HTS for traced sex partners e.g., HIV test kits and gloves. It was seen to have strong sustainability since procurement procedures were well prescribed and supported by the national medical supplies’ reporting tools (**Table 2,** *Fig 3*).

**Table 2:** Quotes comparing integration at baseline and follow-up.

Subsystem	Summary	Baseline interview quotes	Follow-up interview quotes
**Supportive**			
Medical supplies and commodities	aPS was fully integrated into the national HIV supply system at both baseline and follow-up	*P*: *…*.*The county will (support) allocation of the commodities (like) HIV testing kits*. *There we have been actively involved*, *like now during every quarter*, *we review our used commodities*, *the balance remaining*, *and what we should procure for the next three months*. (KII 081 SCASCO, Baseline)	*I*:.* *.* *.* *.*in terms of supplies*, *do you feel that aPS is fully integrated within the KEMSA (Kenya Medical Supplies Agency) supply system P*: *Yes*, *because we do it and*.* *.* *. *we report it in the (MOH) 643*, *we report in (MOH) 731 (medical commodity consumption reports)*, *and these are able to reflect the number of clients that have been tested*. (KII 005 SCASCO, follow-up)
**Production**		* *	* *
Integration of aPS into HTS	At both baseline and follow-up, aPS was considered part of routine service delivery	*I*: *Would you say that aPS is provided by HTS providers on routine basis*, *in other words part of routine delivery*? *P*: *Yeah*, *it is part of their routine delivery*. (KII 078 Facility-in-charge, baseline)	*I*: *Would say aPS is integrated within HTS at the facility level*? *P*: *It is fully integrated because all counsellors are aware of aPS and they practice it*. (KII 004 Nursing Officer-in-charge, follow-up)
**Adaptive**		* *	* *
Sensitization/awareness raising	Though there was greater awareness of assisted partner services (aPS) at follow-up compared to baseline, there were gaps with community level sensitization	*P*: *So far*, *I have little literature or advocacy messages which have been put in place*. *However*, *we have involved the CHWs (Community Health Workers) and try to explain to them*. *But you know aPS covers a wider area*, *so there are still a lot of challenges in advocacy*. *People are not aware*. (KII 087 Facility-in-charge, baseline)	*P*: *Sensitization has been happening for healthcare workers majorly in the facilities but at the community level… I don’t think sensitization for aPS has happened*. (KII 003 SCASCO, follow-up)
**Managerial**		* *	* *
Data reporting tools	At both baseline and follow-up, aPS indicators had only been integrated to data reporting tools at the facility level, but not at the national level.	*I*: *Yeah*, *right now as we speak*, *how is data on personal notification captured from the facility to the national level*? *P*: *It is only captured at the facility*. *I do not have data at the national level*. *I am so keen*.* *.* *.* *.*that we start collecting it at national level*. (KII 068—MOH Policy maker, baseline)	*P*: *As for me*, *the biggest gap is integrating the aPS indicators*, *all the way to the national level*. *I*: *OK*. *P*: *We are collecting aPS indicator data at the facility level*, *but our summary tools which is MoH 362 and MOH 711 in the DHIS (Demographic Health Information System) don’t capture that*. KII 001 Implementing Partner, follow-up)
**Maintenance**		* *	* *
Recruitment	At both baseline and follow-up, the government encountered challenges in hiring HTS providers who would offer aPS. Implementing partner organizations helped to fill in this gap.	*P*:.* *.* *.*In terms of Human Resource*, *mostly it is the (implementing) partners who have really financed (aPS)*.* *.* *..* *.* *.. *in terms of transition*, *we can see how the county talks with the (implementing partner) stakeholders on how we can transition (aPS) into our system of human resource*. (KII 081 SCASCO, baseline)	*I*:.* *.* *.* *.*The MoH asks (implementing) partners to support the HTS providers…because government is not able to quickly respond to paying healthcare workers because they expect an annual budget*. *So basically*, *(implementing) partners usually take up the immediate responsibility of recruitment of providers to provide aPS*. *(*KII 001 Implementing Partner, follow-up)
**Funding** *		* *	* *
Budgets	At baseline, aPS budgets were restricted to the national level with no mention of it at the county level. At follow-up, budgets were part of county annual work plan process.	*I*: *Do you know if the government itself or the Ministry of Health or the county departments of health have allocated any monies or budget lines specifically for the aPS*? *P*: *No*. *You know the Ministry of Health and the county for that matter*, *they basically and majorly depend on the implementing partner organizations*.* *.* *..* *.* *. *So*, *they do not have any funds allocated to any activity like the (aPS)*. *(*KII 080 Implementing partner, baseline)	*P*: *Well*, *when we do the annual work plan*, *we plan for everything*, *we budget for everything*. *But it’s usually very hard to get the finances to carry out those activities*. *I*: *The budgeting process*, *is there any point where that is done jointly between (implementing) partners and the MoH*? *P*: *No I*: *So*, *you run on separate budgets*? *P*: *Yes*. *(*KII 005 SCASCO, follow-up)

*Funding was an emerging theme and was added to the conceptual framework.

At both baseline and follow-up, aPS had been fully integrated into HTS delivery (production subsystem) (**Table 2,** *Fig 3*). It had been routinized as a daily task for all HTS providers and was considered part of their key performance indicators. aPS procedures and metrics were also well defined in the national aPS guidelines indicating strong sustainability of the intervention.

There was partial integration of aPS into the adaptive subsystem (planning, sensitization) at baseline that persisted into follow-up (**Table 2,** *Fig 3*). Although the MOH had finalized on aPS guidelines and training materials, community level sensitization was lagging due to lack of a clear communication strategy. At follow-up, there were well-defined planning cycles including quarterly and annual work plans included in the aPS guidelines indicating strong sustainability. However, the lack of a communication strategy meant that low community awareness persisted.

At baseline and follow-up, there was partial integration of aPS in monitoring, evaluation, and supervision by CASCOs, sub-CASCOs and implementing partner organization staff (managerial subsystem) (**Table 2,** *Fig 3*). There was a failure to integrate aPS indicators into the national HTS register that had just been updated prior to aPS being adopted into the HTS program. aPS indicators were, therefore, collected on separate data collection tools provided by the implementing partner organizations at the facility level and relayed through the counties to the national level, making it difficult to proactively track the status of aPS in the country. Despite this challenge, the presence of clear aPS indicators in the national aPS guidelines indicated strong sustainability with plans for the MOH to revise the HTS registers at a later date.

There was partial integration observed in the recruitment and training of HTS providers (maintenance subsystem) at both baseline and follow-up (**Table 2,** *Fig 3*). At baseline, training of HTS providers on aPS was ongoing largely through support from implementing partner organizations. At the same time, the county governments had halted the hiring of new HTS providers due to funding limitations. At follow-up, refresher training on aPS was ongoing. It also emerged that implementing partner organizations were hiring contract HTS providers with approval from the county governments due to persistent funding challenges at the county level, compromising the sustainability of the intervention.

Funding emerged as a key standalone theme that had not been distinctly outlined in our conceptual framework (**Table 2,** *Fig 3).* At both baseline and follow-up, funding budgets had not been integrated with implementing partner organizations operating their own aPS budgets separate from the government. To facilitate county-wide aPS activities, CASCO / SCASCOs held annual work plan meetings with implementing partner organizations to align on budgetary support for aPS related activities. However, the lack of funding budget for aPS ratified by the government compromised sustainability of the intervention.

### Barriers to aPS integration

Major barriers to aPS integration included human resource constraints, funding gaps, and client concerns with aPS (**Table 3**). First of all, stakeholders reported that hiring restrictions on HTS providers constrained service delivery leading to demotivation among existing providers and poor performance in some facilities. Secondly, they felt that limited government funding led to inadequate mentorship, training, and supervision of aPS compromising the service quality. Lastly, client concerns regarding privacy, confidentiality and safety due to inadequate community sensitization on aPS contributed to low client awareness.

**Table 3:** Barriers and facilitators to aPS integration.

Theme	Details	Quotes
**Barriers**		
Human resource challenges	The main human resource challenges especially hiring gaps.	*I*: *What are some of the gaps that still exist*? *P*:.* *.* *.* *..*Human resource constraints*. *Even as we are implementing aPS*, *we have not been able to put human resource because for us to have aPS in a facility*, *we need like 15 or 20 counselors and nobody is hiring and it is just the same few people who are doing the work*. *(*KII 033 MOH Policy Maker, follow-up)
Funding gaps	The main funding gaps were in providing transport reimbursement	*I*: *Have*.. *you experienced any challenges or heard any challenges expressed by the providers with regards to aPS*? *P*: *Okay*, *they report that some of these clients are coming from very far*.* *.* *..* *.* *. *It is difficult to trace them because the program cannot provide funds for that*. *(*KII 078 Facility-in-charge, baseline)
Client concerns	These included client concerns with privacy, confidentiality, safety and risk of intimate partner violence (IPV)	*P*:.* *.* *..* *.* *..*community acceptance still is low because of the stigma issues*. *I told you there are human rights issues on disclosure*. *…*. *So*, *we really need to talk to the community health volunteers (CHVs) to help us gain entry in that village*. *(*KII 012 Facility-in-charge, follow-up)
**Facilitators**		
Increased resources	These resources include funding, human resources, training, and updated reporting tools	*P*: *Yes*, *if you ask me*, *I think there should be additional allocation of the resources*, *especially on the follow up of the (sexual network) tree*, *especially on the sexual partner*. *(*KII 084 Implementing partner, baseline)
Involve community health volunteers (CHVs)	There was need to increase community awareness and participation in aPS.	*P*: *One thing that I would like to say is that through our peer educators and our community health volunteers (CHVs)*, *we have been able to create awareness*. *We have been able to promote entry into the community*, *in that*, *sometimes it demands that one of the HTS provider would request the CHV to go with her to the community*. *(*KII 085 Facility-in-charge, baseline)

### Facilitators of aPS integration

Stakeholders noted two crucial facilitators to aPS integration (**Table 3**). First, they noted that aPS was resource intensive requiring additional funding and human resources to effectively facilitate partner tracing and notification. Additional resources would go a long way in facilitating aPS integration and service delivery. Secondly, stakeholders thought that community health volunteers (CHVs) could play a pivotal role in increasing community awareness of aPS and supporting HTS providers during partner tracing and notification.

## Discussion

From 2016 to 2018, Kenya had made significant progress in integrating aPS into its national HTS program with varying levels of integration across organizational subsystems. Overall, aPS had become routine practice supported by national guidelines and policies that detailed inputs, processes and procedures implying high sustainability of the intervention. Funding limitations, staff hiring constraints, and low community awareness were key barriers to effective integration. However, increased resource allocation towards aPS-related activities, and strategies to promote community awareness would go a long way in improving its integration.

aPS comes along side several other interventions that have been integrated to HTS programs e.g., tuberculosis (TB) screening, provision of PrEP, and family planning [26,27,31]. In our study, we found that aPS had been fully integrated into procurement and service delivery indicating that partner notification services were being provided to clients. Despite this improved access to aPS, challenges were noted during implementation, similar to other studies. In a systematic review of integrated HIV / TB services, there was improved TB case-finding among clients testing for HIV [31]. Despite the feasibility of this integration, authors encountered programmatic, staffing, infrastructural challenges due to paucity of financial and human resources that negatively impacted the integration process, akin to what we found in our study [32]. MOH policymakers will need to ensure adequate financial support to ensure sustainability of programs even as external funding support declines.

In our study, we found partial integration in recruitment, training, monitoring, evaluation, and supervision of HTS providers (maintenance and managerial subsystems) largely due to limited government funding. Implementing partner organizations, which play a major role supporting aPS budgets, might be hesitant to support budgets to which both the national or county governments have not committed any resources. Similar challenges were noted in family planning integration where human resource challenges, inadequate planning and limited budgets were noted as key impediments [27]. In in Zimbabwe, competing clinical responsibilities and insufficient training budgets were seen as major limitations to family planning integration [33]. Policymakers will need to negotiate with national treasuries to support hiring and training of HTS providers to support aPS and engage contract staff as a stopgap measure in case of limited funding.

We found low community awareness of aPS, similar to a systematic review evaluating the integration of essential family planning and prevention of mother to child transmission interventions to HTS [27]. Participants reported poor access to family planning information with authors recommending the incorporation of female peer educators living with HIV to deliver family planning information to the community. In our study, stakeholders advocated for a two-pronged approach involving: (1) a comprehensive community engagement strategy to improve mass education on both aPS and HIV testing, and (2) an individualized approach offering targeted aPS and HTS to sex partners through HTS providers and CHVs. These approaches will improve awareness on aPS but will require extensive stakeholder consultations on resource allocation against competing health interests e.g., COVID-19 response.

Another program that has recently been integrated to the national HTS program in Kenya is PrEP. In a review of PrEP integration in the country, authors noted service bottlenecks as a challenge to integration with clients initiating PrEP spending an additional 18 minutes on PrEP-specific activities [26]. aPS integration into HTS could potentially lead to such bottlenecks when traced sex partners are referred for further care and treatment at health facilities. Healthcare managers will need to anticipate such constraints and either avail the necessary resources or adapt HTS infrastructure to support additional patients. This may include engaging volunteer HTS providers to elicit, trace, notify, test and link sex partners.

Our study had several strengths. First, we used a conceptual framework combining concepts from program integration, institutionalization, and program sustainability literature, providing a holistic assessment of integration across subsystems. Second, using a subsystems approach, we were able to gain a more granular view of aPS integration. Such an approach will enable stakeholders to more accurately identify integration gaps and develop mitigation plans as appropriate. Third, using qualitative data collected at two time-points, we were able to demonstrate aPS integration over time, highlighting areas with persistent challenges and recommend improvements. Lastly, our purposive sampling approach that included national, county and community level stakeholders enabled a more inclusive view of aPS integration through the different healthcare levels in Kenya.

There are several limitations and methodological challenges in our study. First, our sample size was small compared to those typically used in quantitative studies. However, it was appropriate for qualitative research as it gave an in-depth understanding of stakeholder viewpoints. Second, due to changes in interviewer availability, we engaged two different qualitative researchers at baseline and follow-up which might have affected the consistency of the interviews. However, both interviewers thoroughly reviewed the conceptual framework, interview guides, and codebook before conducting the interviews to ensure uniformity. Third, baseline interviews were conducted at different times because Homa Bay facilities entered the study five months after the start of Kisumu baseline interviews. These temporal differences might have affected the baseline context of aPS integration in the country. Lastly, follow-up interviews were conducted on phone, and not in-person, due to COVID-19 social distancing restrictions which might have led to potential loss of contextual data and compromised probing.

## Conclusion

In summary, our study systematically assessed the integration of aPS into Kenya’s national HTS program. We observed varying degrees of aPS integration across different subsystems highlighting challenges in funding, human resource, and public awareness. To overcome these barriers, we recommend increased resource allocation (funding, human resources) and widespread community-level awareness on aPS. As aPS is being scaled up across many high HIV-burden African countries, further evaluation of national integration efforts and mitigation strategies against implementation gaps is required.

## Supporting information

S1 File Inclusivity questionnaire: PLOS questionnaire on inclusivity in global research. (DOCX)

S2 File Interview guide: Baseline interview guides. (PDF)

S3 File Interview guide: Follow-up interview guides. (PDF)
