# Supplementary material for: Integration of assisted partner services within Kenya’s national HIV testing services program: A qualitative study
Source: PLOS Glob Public Health. 2023 Feb 10;3(2):e0001586. doi: 10.1371/journal.pgph.0001586 (PMC10022023; doi:10.1371/journal.pgph.0001586)
Supplement: S2 File — (PDF) [file pgph.0001586.s002.pdf]

INTERVIEW GUIDE – NASCOP & MOH POLICY-MAKERS AND PLANNERS

Procedure: Study staff will find a suitably private place to meet and to conduct the interview. Thank the participant for agreeing to talk with the staff, and explain that the taping procedure is so we don't miss any important things that he/she says, but that no names or identifying information will be in the final typed-up transcripts of the interview, and the tapes themselves will be destroyed at the latest 5 years after the study completed, hence [dd/mm/yyyy].

Further explain that the "ground rules" for the interview are that every word that is said will be held in the strictest confidence. Explain that we take their confidentiality very seriously and we trust that they will as well. We take the information they share with us very seriously and we expect completely honest answers. If at any time the participant does not wish to answer a question or wants to terminate the interview, they are free to do so.

Study ID Number: _____________________ Participant's Gender: Male □ Female □

Length of session: _______ Participant's Age: ____________

Date: ___/___/____ Starting Time: _________ Primary facilitator: _____________________

Recording device: _____________ Recording digital file #: _______________

General information

- What is your professional designation?
- What is your role in Assisted Partner Notification services (aPNS) implementation?
- How long have you been involved in aPNS working in HIV services?

(Adaptive Subsystem) Planning, awareness raising and sensitization

- What do you know about the current policies and guidelines in place for Assisted Partner Notification services (aPNS)?
  Probes:
  - Is there a specific policy addressing aPNS? If yes, which one?
  - Is aPNS incorporated in the national HIV testing guidelines? If yes, how?
  - Has aPNS been incorporated in the Kenya AIDS strategic framework? If yes, how?
  - What is the current progress in terms of policy and guidelines for aPNS?
    - When did this process begin?
    - If the process is not completed, what are the next steps to ensure completeness?
    - What is the timeline for completion of the process?
  - Who were the leaders and influencers that drove development of this policy?
  - What has catalyzed the development of this policy?
  - What are some of the barriers of policy development around aPNS?
- In what ways are current policy(ies) supportive of aPNS?
  Probes:
  - What are the main aspects of the current policy that support aPNS (e.g. target population, role of health providers, modes of aPNS, integration into existing services)

    - What has helped in attaining this policy support for aPNS?
- What are the main policy gaps for aPNS?
  Probes:
    - What is currently being done to strengthen policy support for aPNS?
    - What are some legal or human right barriers that need to be addressed to facilitate aPNS?
- How are aPNS services coordinated at national level?
    - Is there an office or person assigned to oversee the rollout of PNS? If yes, what are the designated roles of this individual?
    - Is there a taskforce for aPNS?
      If yes, Probes
        - Who are the members of this team?
        - How often do they meet?
        - What are their main responsibilities?
- What plans exist for sensitizing the healthcare workers on aPNS?
    - If not, what is the current status?
    - What is the timeline for development of such a plan to be developed?
- What plans exist for sensitizing the community on aPNS?
    - If not, what is the current status?
    - What is the timeline for such a plan to be developed?

(Supportive) Procurement and logistics

- How is aPNS currently funded?
  Probes
    - Who has currently funded aPNS activities relating to policy development?
    - What are the main funding sources for implementation for policy development and planning at national level?
    - Do implementing partners (PEPFAR and Global Fund) have targets and budgets for aPNS?
    - What plans exist for future funding of aPNS activities?
- What are additional resources that may be required for aPNS delivery?
  Probes:
    - Have resource needs for aPNS been assessed e.g. staff, test kits, partner tracing, airtime, home visits, training?
    - Have the financing requirements for aPNS been assessed?
    - Have government budgets been adjusted to include aPNS?
    - Have county budgets been adjusted to include aPNS?
    - Have implementing partners' targets and budgets been adjusted to include aPNS?
- What modifications, if any, have been made to budgets to include additional expenses for aPNS (e.g., telephone bills, training costs, tracing costs)?
- What adjustments, if any, are needed to the supply chain, procurement and logistics systems to handle the additional supplies needed for aPNS (e.g., additional testing kits)?

(Maintenance) Recruitment

- How is aPNS integrated into routine service delivery?
    - Who conducts aPNS?
    - Who pays for the partner tracing?
    - How is aPNS activities integrated to HTS?

    - What is the current status of aPNS integration?
    - What is the timeline for aPNS integration to occur?
- What changes have been made or are needed in service delivery to ensure aPNS is integrated into HTS?
    - Probe for changes such as phone tracking, change of data tools, rescheduling of community outreaches
- In what ways should staff be reorganized (e.g. adding staff numbers, changing work flows or increasing staff salaries) to accommodate aPNS?
- What modifications, if any, have been made to include aPNS in job descriptions or assigned roles and responsibilities for HTS providers?

(Maintenance) Staff training

- Have training materials for aPNS been developed?
    - If yes,
        - What materials have been developed?
        - Who are these materials targeting?
        - Who is missed out?
    - If no, why not?
- How has aPNS been incorporated into routine training for HTS providers?
- Have SOPs or job aids for HTS providers been developed for aPNS?
- Have performance review or job evaluation processes for HTS providers been modified to include aPNS?
- Have reward, recognition, or promotion systems for HTS providers been modified to include aPNS?

(Managerial) M&E and Supervision

- How is aPNS monitored?
  Probes:
    - How are data on partner notification captured from facility to national level?
    - What changes, if any, have been made to existing data collection systems (e.g. registers) to capture information about aPNS?
        - How are aPNS processes (e.g. contact information, tracking of tracing attempts, linking of index clients to their partners) documented?
    - What is the current status?
    - What are the planned next steps?
    - What is the timeline for that to occur?
- What is the dissemination plan for aPNS guidelines?
  Probes
    - Has policy guidance for aPNS been issued?
    - What is the current status?
    - What is the timeline for that to occur?

Quality assurance for aPNS

- What system, if any, exists for support supervision to deal with peer-to-peer support for aPNS (debriefing)?
- What system exists for mentoring or joint problem solving?
- How is quality assurance review of HIV tests conducted under aPNS?

INTERVIEW GUIDE – aPNS IMPLEMENTING PARTNERS

Procedure: Study staff will find a suitably private place to meet and to conduct the interview. Thank the participant for agreeing to talk with the staff, and explain that the taping procedure is so we don't miss any important things that he/she says, but that no names or identifying information will be in the final typed-up transcripts of the interview, and the tapes themselves will be destroyed at the latest 5 years after the study completed, hence [dd/mm/yyyy].

Further explain that the "ground rules" for the interview are that every word that is said will be held in the strictest confidence. Explain that we take their confidentiality very seriously and we trust that they will as well. We take the information they share with us very seriously and we expect completely honest answers. If at any time the participant does not wish to answer a question or wants to terminate the interview, they are free to do so.

Study ID Number: ____________________ Participant's Gender: Male □ Female □

Length of session: _______ Participant's Age: ____________

Date: ___/___/____ Starting Time: _________ Primary facilitator: _____________________

Recording device: _____________ Recording digital file #: _______________

General information

- What is your professional designation?
- What is your role in Assisted Partner Notification services (aPNS) implementation?
- How long have you been involved in aPNS working in HIV services?

(Adaptive Subsystem) Planning, awareness raising and sensitization

- What do you know about the current policies and guidelines in place for Assisted Partner Notification services (aPNS)?
  Probes:
  - What is the current process in terms of policy and guidelines for aPNS?
  - If the process is not completed, what are the next steps to ensure completeness?
  - What is the timeline for completion of the process?
  - Who were the leaders and influencers that drove development of this policy?
  - What has catalyzed the development of this policy?
  - What are some of the barriers of policy development around aPNS?
- What are the main policy gaps for aPNS?
  Probes:
  - How supportive is the legal framework for delivery of aPNS?
  - If supportive, what has helped in attaining this?
  - If not supportive, what is being done to facilitate aPNS?
  - What are some legal or human right barriers that need to be addressed?

(Supportive) Procurement and logistics

- What are additional resources that may be required for aPNS delivery?

Probes:

- What are the major funding sources for aPNS currently and in the next 5 years?
- How have government budgets been adjusted to include aPNS?
- How have county budgets been adjusted to include aPNS?
- How have implementing partners' targets and budgets been adjusted to include aPNS?

- What adjustments, if any, are needed to procurement and logistics systems to handle the additional supplies needed for aPNS (e.g., additional testing kits)?
- In what ways have budgets been modified to include additional expenses for aPNS (e.g., telephone bills, training costs, tracing costs)?

(Maintenance) Recruitment and training

- How is aPNS integrated into routine service delivery?
  - Who conducts aPNS?
  - Who pays for the partner tracing?
  - Is this integrated to HTS or handled separately as aPNS?
  - What is the current status?
  - What is the timeline for that to occur?
- What changes, if any, have been made in service delivery to ensure aPNS is integrated into HTS?
  - What are those (phone tracking, change of data tools, rescheduling of community outreaches)?
- In what ways should staff be reorganized (e.g. adding staff numbers, changing work flows or increasing staff salaries) to accommodate aPNS?

(Managerial) M&E and Supervision

- How is aPNS monitored?

  Probes:
  - What changes, if any, have been made to existing data collection systems (e.g. registers) to capture information about aPNS?
    - How are the aPNS processes (e.g. contact information, tracking of tracing attempts, linking of index clients to their partners) documented?
  - What are the planned next steps?
  - What is the timeline for that to occur?
- What is the dissemination plan for aPNS guidelines?

  Probes
  - What is the timeline for the dissemination to occur?

Quality assurance for aPNS

- What system exists, if any, for support supervision to deal with peer-to-peer support for aPNS (debriefing)?
  - How about mentoring or joint problem solving; what system exists for mentoring or joint problem solving?
  - How about quality assurance review of HIV tests; what system exists for quality assurance?

INTERVIEW GUIDE – Facility in charges, CASCOs, sub-CASCOs

Procedure: Study staff will find a suitably private place to meet and to conduct the interview. Thank the participant for agreeing to talk with the staff, and explain that the taping procedure is so we don't miss any important things that he/she says, but that no names or identifying information will be in the final typed-up transcripts of the interview, and the tapes themselves will be destroyed at the latest 5 years after the study completed, hence [dd/mm/yyyy].

Further explain that the "ground rules" for the interview are that every word that is said will be held in the strictest confidence. Explain that we take their confidentiality very seriously and we trust that they will as well. We take the information they share with us very seriously and we expect completely honest answers. If at any time the participant does not wish to answer a question or wants to terminate the interview, they are free to do so.

Study ID Number: _____________________ Participant's Gender: Male □ Female □

Length of session: _______ Participant's Age: ____________

Date: ___/___/____ Starting Time: _________ Primary facilitator: _____________________

Recording device: _____________ Recording digital file #: _______________

General information

- What is your professional designation?
- How long have you worked in HIV services at this or other facilities?
- What is your role in Assisted Partner Notification services (aPNS) implementation?
- How long have you been involved in aPNS working in HIV services?

(Adaptive Subsystem) Planning, awareness raising and sensitization

- What steps, if any, have been taken to include aPNS in county work plans and budgets?
- What steps if any, have been taken to create a sense of ownership from county governments?
- What activities are in place to create demand for aPNS services?
    - Are community educators informed or briefed on aPNS to create demand?
    - How is demand for aPNS created? In what ways are health talks used in creating demand for aPNS?
- How are clients tested through aPNS tracked at the facility?
- In which ways are clients tested through aPNS lost to follow-up after contacting them?

Probes: For the preceding questions, probe with the questions: What is the current status? What is the timeline for that to occur?

(Production) PNS Service Delivery

- What is the current status of aPNS service delivery at the facility/sub-county/county?
    - Have HTS providers begun offering aPNS?
        - If so, in which facilities? If no, why not?
    - Are all HTS providers in the facility doing aPNS or not?

            - If no, why not?
            - If yes, on average, how may clients have been offered aPNS at this facility?
            - If yes, are HTS providers offering aPNS to clients routinely?
                - If no, why not?
                - If yes, how have providers incorporated aPNS into their work routine?
                - How well does aPNS fit within providers' work routine?
- What is the future plan for offering aPNS at the facility?
    - What is the future plan for increasing number of facilities/ providers offering aPNS?
    - What is the future plan for integrating aPNS into providers' work routine?
- How is aPNS coordinated with the Dreams program (If available)?
- What are the successes have HTS providers experienced when conducting aPNS?
- What challenges/barriers have HTS providers encountered when conducting aPNS?
- What suggestions or recommendations do you have for improving aPNS integration?
