# Supplementary material for: Integration of assisted partner services within Kenya’s national HIV testing services program: A qualitative study
Source: PLOS Glob Public Health. 2023 Feb 10;3(2):e0001586. doi: 10.1371/journal.pgph.0001586 (PMC10022023; doi:10.1371/journal.pgph.0001586)
Supplement: S3 File — (PDF) [file pgph.0001586.s003.pdf]

INTERVIEW GUIDE – MOH NASCOP POLICY-MAKERS

Procedure: Study staff will find a suitably private place to meet and to conduct the interview. Thank the participant for agreeing to talk with the staff, and explain that the taping procedure is so we don't miss any important things that he/she says, but that no names or identifying information will be in the final typed-up transcripts of the interview, and the tapes themselves will be destroyed at the latest 5 years after the study completed, hence [dd/mm/yyyy].

Further explain that the "ground rules" for the interview are that every word that is said will be held in the strictest confidence. Explain that we take their confidentiality very seriously and we trust that they will as well. We take the information they share with us very seriously and we expect completely honest answers. If at any time the participant does not wish to answer a question or wants to terminate the interview, they are free to do so.

Study ID Number: ______________________ Participant's Gender: Male □ Female □

Length of session: ________ Participant's Age: _____________

Date: ___/___/____ Starting Time: __________ Primary facilitator: ______________________

Recording device: ______________ Recording digital file #: ________________

General information

- What is your professional designation?
- What is your role in Assisted Partner Notification services (aPNS) implementation?
- How long have you been involved in aPNS working in HIV services?

Adaptive subsystem - planning and sensitization

1. **Please tell me about the current laws, guidelines and policies in place for Assisted Partner Notification services (aPNS)?**
   - What is the current status of the laws and guidelines on aPNS?
     Probes:
     - Guidelines
       - Have the aPNS guidelines officially been launched? If yes, when?
       - (If yes) What other guidelines were launched together with the aPNS guidelines?
       - (If mentioned) In your view, in what ways did the joint launch of the aPNS and self-testing guidelines affect messaging on aPNS?
       -
     - Policies
       - What are the main policy gaps for aPNS that need to be addressed?
     - Legal challenges
       - What legal/human rights challenges have been raised since aPNS was scaled-up country-wide? What has been done to address these legal/ human rights challenges?

2. **Please tell me how aPNS is coordinated at the national level?**
   - (Sensitization, service delivery, joint planning, HIV self-testing, joint meetings, TWGs)
   - Who are the key individuals involved at all levels (national, county, sub-county, community)? What are their roles?

- What plans exist for coordination at all levels (national, county, sub-county, community)? Is there a formal work plan for coordination?
- What plans exist for sensitization at all levels (national, county, sub-county, community)? Is there a formal media communication strategy to improve awareness aPNS in the country?

3. **Do you see aPNS planning & sensitization becoming a regular/routine occurrence?**
    - Will aPNS guideline/policy/law review be a one-time occurrence or will it be repeated? If so, how frequently?
    - Will aPNS sensitization be a one-time occurrence or will it be repeated? If so, how frequently?
    - Will aPNS workplan review be a one-time occurrence or will it be repeated? If so, how frequently?

Supportive: procurement and logistics

4. **How is aPNS currently funded at the national level?**
   Probes
    - Are there budgets for aPNS? If yes, who supports these aPNS national budgets?
    - What is your program funding primarily used for – human resource, coordinating TWGs, developing plans, policy reviews?
    - Who sets the aPNS performance targets? Are these pegged to aPNS funding?
    - Is there coordination of budgets at the county levels?
    - Are there any forms of funding coordination between national level, implementing partners, and the counties?
    - What is primarily funded by the national government?
    - What is primarily funded by partners?
    - Will aPNS funding be a one-time occurrence or will it be repeated? If so, how frequently?

5. **Please describe how procurement of aPS supplies is coordinated at the national level?**
    - Where does the supplies come from?
    - Who pays for them?
    - How often are the supplies sent?
    - What is the system of reporting for the supplies?
    - What kind of support has the partners given to the MoH in terms of supplies management?
    - What adjustments, if any, are needed to the national supply chain, procurement and logistics systems to handle the additional supplies needed for aPNS (e.g., additional testing kits)?
    - Will aPNS procurement be a one-time occurrence or will it be repeated? If so, how frequently?

6. **What other factors at the national level do you think facilitate or hinder full integration of aPNS?**

Maintenance: staff recruitment, training

7. **What plans exist at the MoH national/policy level to integrate aPNS into the current county health workforce?**
    - (SOPs in place, joint training plans, joint recruitment plans, drafted and shared circulars/memos on aPNS integration for all care workers)

- Who is currently doing aPNS and why?
- What other cadres are implementing aPNS? e.g. mentor mothers, CHVs, peer educators?
- What plans are there for hiring and training staff? Which cadres will be hired? Who will be trained?
- What plans are there to hire and train HTS/aPS national coordinators? Which cadres will be hired?
- Who is currently paying for aPS health workforce and why?
- Are there plans to revise the MoH health workforce job description to include aPNS delivery?

8. **Do you see aPNS recruitment and training becoming a regular/routine occurrence?**
    - Will aPNS hiring be a one-time occurrence or will it be repeated? If so, how frequently?
    - Will aPNS training be a one-time occurrence or will it be repeated? If so, how frequently?

Managerial: M&E, supervision

9. **Are there any changes that have been made at the national/policy level to ensure aPNS is fully integrated in health care service delivery?**
    - Staffing? In what ways should staff be reorganized (e.g. adding staff numbers, changing work flows or increasing staff salaries) to accommodate aPNS?
    - Are there any challenges HTS National coordinators have outlined as they deliver aPNS? What are these? How have they been addressed?
    - What facilitators have HTS national coordinators outlined for full aPNS service delivery?

10. **Do you see aPNS becoming a regular/routine occurrence in HTS?**
    - If yes, how frequently?

Managerial: M&E, Supervision

11. **Please describe how aPNS data is currently being handled and managed?**
    - What M&E system exist for aPNS activities? Is it integrated into the existing system?
    - What tools are being used? (Paper, electronic)
    - How are the tools integrated with the MoH tools?
    - How is the data collated from different facilities?
    - What system exist to make sure that aPS data it is reaching the national level?
    - Who is handling the data at the implementing partner level?
    - Do you have data quality assessments (DQAs)? How often do they happen? Who conducts the DQAs? Does a standard tool exist?
    - How are aPNS processes (e.g. contact information, tracking of tracing attempts, linking of index clients to their partners) documented?
    - How is the safety of client information assured for both paper and electronic tools? What is the role of NASCOP in this process?

12. **Please describe how aPS staff are being supervised and monitored at the MoH/national level?**
    - Is there support supervision for aPNS? If yes, who is supporting/funding this? If no, why not?
    - What system exists for mentoring or joint problem solving?
    - How is NASCOP involved in this process?
    - What concerns about aPNS are being raised during support supervision – safety, burnout, salaries, need for incentives?

13. **Please describe how quality assurance of aPNS is conducted at the county level?** (Are they integrated into HTS)?
    - In what ways have performance reviews for HTS providers been modified to include aPNS? Are there aPNS specific key performance indicators?
    - How is the quality of phone/physical tracing assured? Who does this?
    - What are the plans to incorporate quality assurance into the current quality improvement for HTS?How is NASCOP involved in this process?

14. **Do you see aPNS M&E and supervision becoming a regular/routine occurrence?**
    - Will aPNS data review be a one-time occurrence or will it be repeated? If so, how frequently?
    - Will aPNS performance review be a one-time occurrence or will it be repeated? If so, how frequently?
    - Will aPNS counsellor supervision be a one-time occurrence or will it be repeated? If so, how frequently?

Conclusion

15. **Any other comments do you have on aPNS in Kenya?**
    - What recommendations would you make on aPNS in Kenya?
    - Are there other gaps we have not yet discussed?
    - Are there other populations that should receive aPNS e.g. truck drivers, key populations, fisher-folk?
    - Are there other service delivery points where aPNS should be delivered e.g. blood banks, TB clinics, STI clinics? How would you propose it be delivered?

INTERVIEW GUIDE – aPNS IMPLEMENTING PARTNERS

Procedure: Study staff will find a suitably private place to meet and to conduct the interview. Thank the participant for agreeing to talk with the staff, and explain that the taping procedure is so we don't miss any important things that he/she says, but that no names or identifying information will be in the final typed-up transcripts of the interview, and the tapes themselves will be destroyed at the latest 5 years after the study completed, hence [dd/mm/yyyy].

Further explain that the "ground rules" for the interview are that every word that is said will be held in the strictest confidence. Explain that we take their confidentiality very seriously and we trust that they will as well. We take the information they share with us very seriously and we expect completely honest answers. If at any time the participant does not wish to answer a question or wants to terminate the interview, they are free to do so.

Study ID Number: ____________________ Participant's Gender: Male □ Female □

Length of session: _______ Participant's Age: ____________

Date: ___/___/____ Starting Time: _________ Primary facilitator: _____________________

Recording device: _____________ Recording digital file #: _______________

General information

- What is your professional designation?
- What is your role in Assisted Partner Notification services (aPNS) implementation?
- How long have you been involved in aPNS working in HIV services?

Adaptive subsystem - planning and sensitization

1. **Please tell me about the current laws, guidelines and policies in place for Assisted Partner Notification services (aPNS)?**
   - What is the current status of the laws and guidelines on aPNS?
     Probes:
   - Guidelines
     - Have the aPNS guidelines officially been launched? If yes, when?
     - (If yes) What other guidelines were launched together with the aPNS guidelines?
     - (If mentioned) In your view, in what ways did the joint launch of the aPNS and self-testing guidelines affect messaging on aPNS?
     -
   - Policies
     - What are the main policy gaps for aPNS that need to be addressed?
   - Legal challenges
     - What legal/human rights challenges have been raised since aPNS was scaled-up country-wide? What has been done to address these legal/ human rights challenges?

2. **Please tell me how aPNS is coordinated at the partner level?**
   - (Sensitization, service delivery, joint planning, HIV self-testing, joint meetings, TWGs)
   - Who are the key individuals involved at all levels? What are their roles?
   - What plans exist for coordination at levels you are aware of – whether national, county, sub-county, community? Is there a formal work plan for coordination?

- What plans exist for sensitization at the levels you are aware of – whether national, county, sub-county, community?

3. **Do you see aPNS planning & sensitization becoming a regular/routine occurrence?**
    - Will aPNS sensitization be a one-time occurrence or will it be repeated? If so, how frequently?
    - Will aPNS workplan review be a one-time occurrence or will it be repeated? If so, how frequently?

Supportive: procurement and logistics

4. **How is aPNS currently funded at the implementing partner level?**
   Probes
    - Are there budgets for aPNS? If yes, who supports these aPNS partner budgets?
    - What is your program funding primarily used for – human resource, partner tracing?
    - Who sets the aPNS performance targets? Are these pegged to aPNS funding?
    - Is there a joint budget with the county government?
    - Are there any forms of funding coordination between partners and the counties?
    - What is primarily funded by the counties?
    - What modifications, if any, have been made to budgets to include additional expenses for aPNS (e.g., telephone bills, training costs, tracing costs)?
    - Will aPNS funding be a one-time occurrence or will it be repeated? If so, how frequently?

5. **Please describe how procurement of aPNS supplies is done at the implementing level?**
    - Where does the supplies come from? Who pays for them?
    - How often do you receive the supplies?
    - What is the system of reporting for the supplies?
    - What adjustments, if any, are needed to the supply chain, procurement and logistics systems to handle the additional supplies needed for aPNS (e.g., additional testing kits)?
    - Is there an aPNS procurement cycle at the county level e.g. annual review of HTS supplies to support aPNS? If yes, what does it entail?
    - What kind of support has the partners given to the county in terms of supplies?
    - Will aPNS procurement be a one-time occurrence or will it be repeated? If so, how frequently?

6. **What other factors at the county level do you think facilitate or hinder full integration of aPNS?**

Maintenance: staff recruitment, training

7. **What plans exist at the implementing level to integrate aPNS into the current county health workforce?**
    - (SOPs in place, joint training plans, joint recruitment plans, drafted and shared circulars/memos on aPNS integration for all care workers)
    - Who is currently doing aPNS and why?
    - Who is currently paying for aPS health workforce and why?
    - What other cadres are implementing aPNS? e.g. mentor mothers, CHVs, peer educators?
    - What plans are there for hiring and training staff? Which cadres will be hired? Who will be trained?
    - What revisions have been made to the health workforce JDs to include aPNS delivery?

8. **Do you see aPNS recruitment and training becoming a regular/routine occurrence?**
    - Will aPNS hiring be a one-time occurrence or will it be repeated? If so, how frequently?
    - Will aPNS training be a one-time occurrence or will it be repeated? If so, how frequently?

Production: Service delivery

9. **Are there any changes that have been made at the implementing level to ensure aPNS is fully integrated in health care service delivery?**
    - Probe for changes such as phone tracking, change of data tools, rescheduling of community outreaches
    - Staffing? In what ways should staff be reorganized (e.g. adding staff numbers, changing work flows or increasing staff salaries) to accommodate aPNS?
    - What challenges have HTS providers outlined as they deliver aPNS? How have these challenges been addressed? (e.g. safety concerns, legal concerns, potential emotional/physical abuse from traced partners)
    - What facilitators have HTS providers outlined during aPNS service delivery? (E.g. time to trace, airtime; transport)

10. **Do you see aPNS becoming a regular/routine occurrence in HTS?**
    - If yes, how frequently?

Managerial: M&E, supervision

11. **Please describe how aPNS data is currently being handled and managed at the county level**
    - What M&E system exist for aPNS activities? Is it integrated into the existing system?
    - What tools are being used? (Paper, electronic)
    - How are the tools integrated with the MoH tools?
    - How is the data collected from different facilities?
    - What system exist to make sure data is reaching the national level?
    - Who is handling the data at the county level?
    - Do you have DQAs? How often do they happen? Who conducts the DQAs? Does a standard DQA tool exist?
    - How are aPNS processes (e.g. contact information, tracking of tracing attempts, linking of index clients to their partners) documented?
    - How is the safety of client information assured for both paper and electronic tools?

12. **Please describe how aPS staff are being supervised and monitored at the county level?**
    - Is there counsellor support supervision for aPNS? If yes, who is supporting/funding this? If no, why not?
    - What system exists for mentoring or joint problem solving?
    - What concerns about aPNS are being raised during support supervision – safety, burnout, salaries, need for incentives?
    - How is the county involved in this process?

13. **Please describe how quality assurance of aPNS conducted at the county level?** (Are they integrated into HTS)?
    - In what ways have performance reviews for HTS providers been modified to include aPNS? Are there aPNS specific key performance indicators?
    - How is the quality of phone/physical tracing assured? Who does this?

- What are the plans to incorporate quality assurance into the current quality improvement for HTS?
- How is the county involved in this process?

14. Do you see aPNS M&E and supervision becoming a regular/routine occurrence?

- Will aPNS data review be a one-time occurrence or will it be repeated? If so, how frequently?
- Will aPNS performance review be a one-time occurrence or will it be repeated? If so, how frequently?
- Will aPNS counsellor supervision be a one-time occurrence or will it be repeated? If so, how frequently?

Conclusion

15. Any other comments do you have on aPNS in Kenya?

- What recommendations would you make on aPNS in Kenya?
- Are there other gaps we have not yet discussed?
- Are there other populations that should receive aPNS e.g. truck drivers, key populations, fisher-folk?
- Are there other service delivery points where aPNS should be delivered e.g. blood banks, TB clinics, STI clinics? How would you propose it be delivered?

INTERVIEW GUIDE – CASCOS, SUB-CASCOS

Procedure: Study staff will find a suitably private place to meet and to conduct the interview. Thank the participant for agreeing to talk with the staff, and explain that the taping procedure is so we don't miss any important things that he/she says, but that no names or identifying information will be in the final typed-up transcripts of the interview, and the tapes themselves will be destroyed at the latest 5 years after the study completed, hence [dd/mm/yyyy].

Further explain that the "ground rules" for the interview are that every word that is said will be held in the strictest confidence. Explain that we take their confidentiality very seriously and we trust that they will as well. We take the information they share with us very seriously and we expect completely honest answers. If at any time the participant does not wish to answer a question or wants to terminate the interview, they are free to do so.

Study ID Number: ______________________ Participant's Gender: Male □ Female □

Length of session: ________ Participant's Age: _____________

Date: ___/___/____ Starting Time: _________ Primary facilitator: ______________________

Recording device: ______________ Recording digital file #: ________________

General information

- What is your professional designation?
- What is your role in Assisted Partner Notification services (aPNS) implementation?
- How long have you been involved in aPNS working in HIV services?

Adaptive subsystem - planning and sensitization

1. **Please tell me about the current laws, guidelines and policies in place for Assisted Partner Notification services (aPNS)?**
    - What is the current status of the laws and guidelines on aPNS?
      Probes:
      - Guidelines
        - Have the aPNS guidelines officially been launched? If yes, when?
        - (If yes) What other guidelines were launched together with the aPNS guidelines?
        - (If mentioned) In your view, in what ways did the joint launch of the aPNS and self-testing guidelines affect messaging on aPNS?
        -
      - Policies
        - What are the main policy gaps for aPNS that need to be addressed?
      - Legal challenges
        - What legal/human rights challenges have been raised since aPNS was scaled-up country-wide? What has been done to address these legal/ human rights challenges?
2. **Please tell me how aPNS is integrated into HTS at the county level?**
    - (sensitization, service delivery, joint planning, HIV self-testing Testing, joint meetings)
    - Who are the key individuals involved at the county level? What are their roles?
    - What plans exist for coordination at levels you are aware of – whether national, county, sub-county, community? Is there a formal work plan for coordination?

- What plans exist for sensitization at the levels you are aware of – whether national, county, sub-county, community?
- What plans exist for sensitizing the health care workers?

3. **Do you see aPNS planning & sensitization becoming a regular/routine occurrence?**
   - Will aPNS sensitization be a one-time occurrence or will it be repeated? If so, how frequently?
   - Will aPNS workplan review be a one-time occurrence or will it be repeated? If so, how frequently?

Supportive: procurement and logistics

4. **How is aPNS currently funded at the county level?**
   - Are there county budgets for aPNS? If yes, who supports these aPNS county budgets?
   - What is the county funding primarily used for – human resource, partner tracing?
   - Who pays for the partner tracing?
   - Who sets the county aPNS performance targets? Are these pegged to aPNS funding?
   - What modifications, if any, have been made to budgets to include additional expenses for aPNS (e.g., telephone bills, training costs, tracing costs)?
   - Will aPNS funding be a one-time occurrence or will it be repeated? If so, how frequently?

5. **Please describe how procurement of aPNS supplies is done at the county level?**
   - Where does the supplies come from? Who pays for them? How often do you receive the supplies?
   - What is the system of reporting for the supplies?
   - What adjustments, if any, are needed to the supply chain, procurement and logistics systems to handle the additional supplies needed for aPNS (e.g., additional testing kits)?
   - Will aPNS procurement be a one-time occurrence or will it be repeated? If so, how frequently?

6. **What other factors at the county level do you think facilitate or hinder full integration of aPNS?**

Maintenance: staff recruitment, training

7. **What plans exist at the county level to integrate aPNS into the current health workforce?**
   - (SOPs in place, training plans, recruitment plans, circulars/memos on aPNS integration for all care workers)
   - Who is currently doing aPNS and why?
   - Who is currently paying for aPS health workforce and why?
   - What other cadres are implementing aPNS? e.g. mentor mothers, CHVs, peer educators?
   - What plans are there for hiring and training staff? Which cadres will be hired? Who will be trained?
   - What revisions have been made to the health workforce JDs to include aPNS delivery?

8. **Do you see aPNS recruitment and training becoming a regular/routine occurrence?**
   - Will aPNS hiring be a one-time occurrence or will it be repeated? If so, how frequently?
   - Will aPNS training be a one-time occurrence or will it be repeated? If so, how frequently?

Production: aPNS service delivery

9. **Are there any changes that have been made at the county level to ensure aPNS is fully integrated in health care service delivery?**
    - Probe for changes such as phone tracking, change of data tools, rescheduling of community outreaches
    - Staffing? In what ways should staff be reorganized (e.g. adding staff numbers, changing work flows or increasing staff salaries) to accommodate aPNS?
    - What challenges have HTS providers outlined as they deliver aPNS? How have these challenges been addressed? (e.g. safety concerns, legal concerns, potential emotional/physical abuse from traced partners)
    - What facilitators have HTS providers outlined during aPNS service delivery? (E.g. time to trace, airtime; transport)

10. **Do you see aPNS becoming a regular/routine occurrence in HTS?**
    - If yes, how frequently?

Managerial: M&E and Supervision

11. **Please describe how aPNS data is currently being handled and managed at the county level**
    - What M&E system exist for aPNS activities? Is it integrated into the existing system?
    - What tools are being used? (Paper, electronic)
    - How are the tools integrated with the MoH tools?
    - How is the data collected from different facilities?
    - What system exist to make sure data is reaching the national level?
    - Who is handling the data at the county level?
    - Do you have DQAs? How often do they happen? Who conducts the DQAs? Does a standard DQA tool exist?
    - How are aPNS processes (e.g. contact information, tracking of tracing attempts, linking of index clients to their partners) documented?
    - How is the safety of client information assured for both paper and electronic tools?

12. **Please describe how aPS staff are being supervised and monitored at the county level?**
    - Is there counsellor support supervision for aPNS? If yes, who is supporting/funding this? If no, why not?
    - What system exists for mentoring or joint problem solving?
    - What concerns about aPNS are being raised during support supervision – safety, burnout, salaries, need for incentives?
    - How is the county involved in this process?

13. **Please describe how quality assurance of aPNS conducted at the county level?** (Are they integrated into HTS)?
    - In what ways have performance reviews for HTS providers been modified to include aPNS? Are there aPNS specific key performance indicators?
    - How is the quality of phone/physical tracing assured? Who does this?
    - What are the plans to incorporate quality assurance into the current quality improvement for HTS?
    - How is the county involved in this process?

14. **Do you see aPNS M&E and supervision becoming a regular/routine occurrence?**

- Will aPNS data review be a one-time occurrence or will it be repeated? If so, how frequently?
- Will aPNS performance review be a one-time occurrence or will it be repeated? If so, how frequently?
- Will aPNS counsellor supervision be a one-time occurrence or will it be repeated? If so, how frequently?

Conclusion

15. Any other comments do you have on aPNS in Kenya?

- What recommendations would you make on aPNS in Kenya?
- Are there other gaps we have not yet discussed?
- Are there other populations that should receive aPNS e.g. truck drivers, key populations, fisher-folk?
- Are there other service delivery points where aPNS should be delivered e.g. blood banks, TB clinics, STI clinics? How would you propose it be delivered?

INTERVIEW GUIDE –FACILITY IN CHARGES

Procedure: Study staff will find a suitably private place to meet and to conduct the interview. Thank the participant for agreeing to talk with the staff, and explain that the taping procedure is so we don't miss any important things that he/she says, but that no names or identifying information will be in the final typed-up transcripts of the interview, and the tapes themselves will be destroyed at the latest 5 years after the study completed, hence [dd/mm/yyyy].

Further explain that the "ground rules" for the interview are that every word that is said will be held in the strictest confidence. Explain that we take their confidentiality very seriously and we trust that they will as well. We take the information they share with us very seriously and we expect completely honest answers. If at any time the participant does not wish to answer a question or wants to terminate the interview, they are free to do so.

Study ID Number: _____________________ Participant's Gender: Male □ Female □

Length of session: _______ Participant's Age: ____________

Date: ___/___/____ Starting Time: _________ Primary facilitator: _____________________

Recording device: _____________ Recording digital file #: _______________

General information

- What is your professional designation?
- What is your role in Assisted Partner Notification services (aPNS) implementation?
- How long have you been involved in aPNS working in HIV services?

Adaptive Subsystem - Planning and Sensitization

1. **Please tell me about the current laws, guidelines and policies in place for Assisted Partner Notification services (aPNS)?**
    - What is the current status of the laws and guidelines on aPNS?

    Probes:
    - <u>Guidelines</u>
        - Have the aPNS guidelines officially been launched? If yes, when?
        - (If yes) What other guidelines were launched together with the aPNS guidelines?
        - (If mentioned) In your view, in what ways did the joint launch of the aPNS and self-testing guidelines affect messaging on aPNS?
        -
    - <u>Policies</u>
        - What are the main policy gaps for aPNS that need to be addressed?
    - <u>Legal challenges</u>
        - What legal/human rights challenges have been raised since aPNS was scaled-up country-wide? What has been done to address these legal/ human rights challenges?

2. **Please tell me how aPNS is integrated into HTS at the facility level?**
    - (Sensitization, service delivery, joint planning, HIV self-testing, joint meetings)
    - Who are the key individuals involved at the facility level? What are their roles?
    - What plans exist for sensitizing the health care workers?
    - What plans exist for sensitizing the community?

3. **Do you see aPNS planning & sensitization becoming a regular/routine occurrence?**
    - Will aPNS sensitization be a one-time occurrence or will it be repeated? If so, how frequently?
    - Will aPNS workplan review be a one-time occurrence or will it be repeated? If so, how frequently?

Supportive: Procurement and Logistics

4. **How is aPNS currently funded at the facility level?**
    - Are there facility budgets for aPNS? If yes, who supports these aPNS facility budgets?
    - What is the facility funding primarily used for – human resource, partner tracing?
    - Who pays for the partner tracing?
    - Who sets the facility aPNS performance targets? Are these pegged to aPNS funding?
    - What modifications, if any, have been made to budgets to include additional expenses for aPNS (e.g., telephone bills, training costs, tracing costs)?
    - Will aPNS funding be a one-time occurrence or will it be repeated? If so, how frequently?

5. **Please describe how procurement of aPNS supplies is done at the facility level?**
    - Where does the supplies come from? Who pays for them? How often do you receive the supplies?
    - What is the system of reporting for the supplies?
    - What adjustments, if any, are needed to the supply chain, procurement and logistics systems to handle the additional supplies needed for aPNS (e.g., additional testing kits)?
    - Will aPNS procurement be a one-time occurrence or will it be repeated? If so, how frequently?

6. **What other factors at the facility level do you think facilitate or hinder full integration of aPNS?**

Maintenance: Staff Recruitment, Training

7. **What plans exist at the facility level to integrate aPNS into the current health workforce?**
    - (SOPs in place, training plans, recruitment plans, circulars/memos on aPNS integration for all care workers)
    - Who is currently doing aPNS and why?
    - What other cadres are implementing aPNS? e.g. mentor mothers, CHVs, peer educators?
    - What plans are there for hiring and training staff? Which cadres will be hired? Who will be trained?
    - What revisions have been made to the health workforce JDs to include aPNS delivery?

8. **Do you see aPNS recruitment and training becoming a regular/routine occurrence?**
    - Will aPNS hiring be a one-time occurrence or will it be repeated? If so, how frequently?
    - Will aPNS training be a one-time occurrence or will it be repeated? If so, how frequently?

Production: aPS Service Delivery

9. **Are there any changes that have been made at the facility level to ensure aPNS is fully integrated in health care service delivery?**

- Probe for changes such as phone tracking, change of data tools, rescheduling of community outreaches
- Staffing? In what ways should staff be reorganized (e.g. adding staff numbers, changing work flows or increasing staff salaries) to accommodate aPNS?
- What challenges have HTS providers outlined as they deliver aPNS? How have these challenges been addressed? (e.g. safety concerns, legal concerns, potential emotional/physical abuse from traced partners)
- What facilitators have HTS providers outlined during aPNS service delivery? (E.g. time to trace, airtime; transport)

10. Do you see aPNS becoming a regular/routine occurrence in HTS?
- If yes, how frequently?

Managerial: M&E, Supervision

11. Please describe how aPNS data is currently being handled and managed at the facility level
- What M&E system exist for aPNS activities? Is it integrated into the existing system?
- What tools are being used? (Paper, electronic)
- How are the tools integrated with the MoH tools?
- How is the data collected from different facilities?
- What system exist to make sure data is reaching the national level?
- Who is handling the data at the facility level?
- Do you have DQAs? How often do they happen? Who conducts the DQAs? Does a standard DQA tool exist?
- How are aPNS processes (e.g. contact information, tracking of tracing attempts, linking of index clients to their partners) documented?
- How is the safety of client information assured for both paper and electronic tools?

12. Please describe how aPS staff are being supervised and monitored at the facility level?
- Is there counsellor support supervision for aPNS? If yes, who is supporting/funding this? If no, why not?
- What system exists for mentoring or joint problem solving?
- What concerns about aPNS are being raised during support supervision – safety, burnout, salaries, need for incentives?

13. Please describe how quality assurance of aPNS conducted at the facility level? (Are they integrated into HTS)?
- In what ways have performance reviews for HTS providers been modified to include aPNS? Are there aPNS specific key performance indicators?
- How is the quality of phone/physical tracing assured? Who does this?
- What are the plans to incorporate quality assurance into the current quality improvement for HTS?

14. Do you see aPNS M&E and supervision becoming a regular/routine occurrence?
- Will aPNS data review be a one-time occurrence or will it be repeated? If so, how frequently?
- Will aPNS performance review be a one-time occurrence or will it be repeated? If so, how frequently?
- Will aPNS counsellor supervision be a one-time occurrence or will it be repeated? If so, how frequently?

Conclusion

15. Any other comments do you have on aPNS in Kenya?

- What recommendations would you make on aPNS in Kenya?
- Are there other gaps we have not yet discussed?
- Are there other populations that should receive aPNS e.g. truck drivers, key populations, fisher-folk?
- Are there other service delivery points where aPNS should be delivered e.g. blood banks, TB clinics, STI clinics? How would you propose it be delivered?

INTERVIEW GUIDE – COMMUNITY STAKEHOLDER

Procedure: Study staff will find a suitably private place to meet and to conduct the interview. Thank the participant for agreeing to talk with the staff, and explain that the taping procedure is so we don't miss any important things that he/she says, but that no names or identifying information will be in the final typed-up transcripts of the interview, and the tapes themselves will be destroyed at the latest 5 years after the study completed, hence [dd/mm/yyyy].

Further explain that the "ground rules" for the interview are that every word that is said will be held in the strictest confidence. Explain that we take their confidentiality very seriously and we trust that they will as well. We take the information they share with us very seriously and we expect completely honest answers. If at any time the participant does not wish to answer a question or wants to terminate the interview, they are free to do so.

Study ID Number: ____________________ Participant's Gender: Male □ Female □

Length of session: _______ Participant's Age: ____________

Date: ___/___/____ Starting Time: _________ Primary facilitator: ____________________

Recording device: _____________ Recording digital file #: _______________

General information

- What is your professional designation?
- What is your role in Assisted Partner Notification services (aPNS) implementation?
- How long have you been involved in aPNS working in HIV services?

Adaptive Subsystem – Planning, Sensitization

1. **What do you know about the current policies in place for Assisted Partner Notification services (aPNS) for HIV?**
    Probes:
    - Policies
        - What are the main policy gaps for aPNS that need to be addressed?
    - Legal challenges
        - What legal/human rights challenges have been raised since aPNS was scaled-up country-wide? What has been done to address these legal/ human rights challenges?
2. **Please tell me how aPNS is integrated into HTS at the community level?**
    - (Sensitization, service delivery, joint planning, HIV self-testing, joint meetings)
    - Who are the key individuals involved at the community level? What are their roles?
    - What plans exist for sensitizing the health care workers?
    - What plans exist for sensitizing the community?

3. **Do you see aPNS planning & sensitization becoming a regular/routine occurrence?**
    - Will aPNS sensitization be a one-time occurrence or will it be repeated? If so, how frequently?
    - Will aPNS workplan review be a one-time occurrence or will it be repeated? If so, how frequently?

Supportive: Procurement, Logistics

4. **How is aPNS currently funded at the community level?**

- Are there community budgets for aPNS? If yes, who supports these aPNS community budgets?
- What is the community funding primarily used for – sensitization?
- Who pays for the partner tracing?
- Who sets the community aPNS performance targets? Are these pegged to aPNS funding?
- What modifications, if any, have been made to budgets to include additional expenses for aPNS (e.g., telephone bills, training costs, tracing costs)?
- Will aPNS funding be a one-time occurrence or will it be repeated? If so, how frequently?

5. **Please describe how procurement of aPNS supplies is done at the community level?**
    - Where does the supplies come from? Who pays for them? How often do you receive the supplies?
    - What is the system of reporting for the supplies?
    - What adjustments, if any, are needed to the supply chain, procurement and logistics systems to handle the additional supplies needed for aPNS (e.g., additional testing kits)?
    - Will aPNS procurement be a one-time occurrence or will it be repeated? If so, how frequently?

6. **What other factors at the community level do you think facilitate or hinder full integration of aPNS?**

Maintenance: Staff Recruitment, Training

7. **How has aPNS been integrated into staff activities?**
    - Who conducts aPNS at the community level?
    - Have other cadres apart from HTS providers been included e.g. CHVs, mentor mothers, peer educators?
8. **Do you see aPNS recruitment and training becoming a regular/routine occurrence?**
    - Will aPNS hiring be a one-time occurrence or will it be repeated? If so, how frequently?
    - Will aPNS training be a one-time occurrence or will it be repeated? If so, how frequently?

Production: aPNS Service Delivery

9. **What changes have been made or are needed in service delivery to ensure aPNS is integrated into HTS at the community level?**
    - In what ways should the community be reorganized to accommodate aPNS (e.g. more community outreaches, more media campaigns, adding staff numbers, changing work flows or increasing staff salaries)?
    - Are there any challenges <u>community members</u> have outlined with aPNS? What are these? How have they been addressed? (e.g. lack of awareness on aPNS, safety concerns, legal concerns, relationship dissolution, potential emotional/physical abuse from sexual partners)
    - What facilitators have <u>community members</u> outlined during aPNS service delivery? (E.g. community awareness campaigns)?
    - Are there any challenges <u>community-level staff</u> have outlined as they deliver aPNS? What are these? How have they been addressed? (e.g. safety concerns, legal concerns, potential emotional/physical abuse from traced partners)
    - What facilitators have <u>community-level staff</u> outlined during aPNS service delivery? (E.g. time to trace, airtime; transport)

10. Do you see aPNS becoming a regular/routine occurrence in HTS?
- If yes, how frequently?

Managerial: M&E, Supervision

11. Please describe how aPNS data is currently being handled and managed at the community level?
- What M&E system exist for aPNS activities? Is it integrated into the existing system?
- What tools are being used? (Paper, electronic)
- How are the tools integrated with the MoH tools?
- How is the data collected from different facilities?
- What system exist to make sure data is reaching the national level?
- Who is handling the data at the community level?
- Do you have DQAs? How often do they happen? Who conducts the DQAs? Does a standard DQA tool exist?
- How are aPNS processes (e.g. contact information, tracking of tracing attempts, linking of index clients to their partners) documented?
- How is the safety of client information assured for both paper and electronic tools?

12. Please describe how aPS staff are being supervised and monitored at the community level?
- Is there counsellor support supervision for aPNS? If yes, who is supporting/funding this? If no, why not?
- What system exists for mentoring or joint problem solving?
- What concerns about aPNS are being raised during support supervision – safety, burnout, salaries, need for incentives?

13. Please describe how quality assurance of aPNS conducted at the community level? (Are they integrated into HTS)?
- In what ways have performance reviews for HTS providers been modified to include aPNS? Are there aPNS specific key performance indicators?
- How is the quality of phone/physical tracing assured? Who does this?
- What are the plans to incorporate quality assurance into the current quality improvement for HTS?

14. Do you see aPNS M&E and supervision becoming a regular/routine occurrence?
- Will aPNS data review be a one-time occurrence or will it be repeated? If so, how frequently?
- Will aPNS performance review be a one-time occurrence or will it be repeated? If so, how frequently?
- Will aPNS counsellor supervision be a one-time occurrence or will it be repeated? If so, how frequently?

Conclusion

15. Any other comments do you have on aPNS in Kenya?
- What recommendations would you make on aPNS at the community level?
- Are there other gaps we have not yet discussed?
- Are there other populations that should receive aPNS e.g. truck drivers, key populations, fisher-folk?
- Are there other service delivery points where aPNS should be delivered e.g. blood banks, TB clinics, STI clinics? How would you propose it be delivered?
